# Exploring social determinants of disability among older filipinos: insights from a polysocial score approach

**DOI:** 10.1186/s41256-025-00453-7

**Published:** 2025-10-29

**Authors:** Jianhong Xu, Haolin Li, Grace T. Cruz, Yasuhiko Saito, Chenkai Wu

**Affiliations:** 1 Global Health Research Center, Duke Kunshan University, No. 8 Duke Avenue, Kunshan, 215316 Jiangsu China; 2 Population Institute, University of Philippines, Diliman, Quezon City, Philippines; 3 College of Economics, Nihon University, Tokyo, Japan

**Keywords:** Social determinants, Activities of daily living disability, Polysocial score, Older adults

## Abstract

**Background:**

The rapid aging in the Philippines presents significant challenges, including high rates of activities of daily living (ADL) disability among older adults. While research has identified various social determinants of ADL disability, there is a gap in understanding how their joint impact on disability among older Filipinos. We adopted the polysocial score approach to assess these joint associations of multiple social factors with disability among older adults in the Philippines.

**Methods:**

Individuals included in the analysis were from the Longitudinal Study of Ageing and Health in the Philippines. Twenty-nine social factors from five domains were included. The Light Gradient Boosting Machine (LightGBM) model was employed to identify and rank key social determinants, which were then incorporated into a logistic regression model to derive the polysocial score, both continuously and in categories and assessed its association with ADL disability. Model performances were assessed by discrimination, calibration, and reclassification (compared to a reference model). All analyses were separated for men and women.

**Results:**

We included 5,000 participants (37.0% men) with an average age of 68.3 years. The polysocial score included 19 social factors for men and 18 for women. Among men, the most significant predictors were the frequency of engaging in social activities and the number of friends they had contact with. Attending religious services outside the home and the frequency of attending social activities were the most important factors for women. Eleven social factors overlapped between men and women. Individuals with moderate or high polysocial scores exhibited reduced likelihood of experiencing ADL disability relative to those with low scores. We observed satisfactory model performance among men and women.

**Conclusions:**

We identified important social factors for men and women and their joint association with ADL disability. The polysocial score could be used to design person-centered social interventions that promote health and support independence.

**Supplementary Information:**

The online version contains supplementary material available at 10.1186/s41256-025-00453-7.

## Introduction

Although the Philippines has not yet entered an aging society, its population is aging rapidly, as in many other developing countries [1, 2]. By 2055, the proportion of the population aged 60 or over is projected to nearly double, increasing from 8.5% in 2020 to 19.6% [3]. Over one in five older adults in the Philippines experiences disability in activities of daily living (ADLs), such as eating, dressing, and bathing—a prevalent syndrome in geriatric populations [4]. Among older adults, the presence of disability was associated with shorter life expectancy, more frequent hospital admissions, and greater medical expenses, imposing heavy burdens on caregivers and health services [5, 6].

Previous studies have identified several social determinants of ADL disability, such as architectural barriers, communication barriers, social barriers, and poor relationships with relatives [7–10], as well as broader social risk factors including education, wealth, insurance type, marital status, and living arrangements [10–13]. Despite their importance, these social determinants remain understudied among the older population in the Philippines. The current body of literature has not adequately explored the combined effects of multiple social factors on disability among this population.

The recently developed polysocial score approach presents a promising method for quantifying these cumulative influences and has shown potential in various health outcomes [14–17]. By capturing the combined effects of genetic risk, social environment, and lifestyle, the polysocial score offers meaningful insights into shaping health in older adults [16, 18]. Additionally, it offers valuable insights into explaining health disparities [19]. This approach may provide critical insights into the social determinants of ADL disability among older Filipinos, an area that remains notably underexplored.

We aimed to construct and validate a polysocial score for assessing the likelihood of ADL disability among older adults in the Philippines. Because gender differences existed in the level of involvement in social activities [20] and the possibility that the relationship between polysocial score and ADL disability may vary by gender, we conducted gender-stratified analyses to explore differences between men and women. Findings from this study would offer meaningful insights into how social factors impact ADL disability across genders in the Filipino older population and could guide the design of tailored, person-centered social interventions to promote healthy aging and functional independence.

## Methods

### Data source and study design

Data were from the baseline wave of the Longitudinal Study of Ageing and Health in the Philippines (LSAHP)—the country’s first nationwide longitudinal study focused on aging. Employing a multistage sampling design, the study selected provinces as primary sampling units, barangays as secondary units, and individuals aged 60 and above as the final units of selection. To ensure adequate representation of the oldest age groups, respondents aged 70–79 and 80 + were oversampled by factors of two and three, respectively. The LSAHP provided a nationally representative data on the health, socioeconomic status, and general well-being of older adults in the Philippines [21]. The baseline survey was conducted between October 2018 and February 2019 and covered 5,985 respondents with a response rate of 94%. The ethical approval was obtained from the University of the Philippines Manila Research Ethics Board Review Panel 2. All participants provided informed consent prior to their involvement in the LSAHP. Details of the study design and recruitment strategies have been provided elsewhere [4]. We used baseline data and excluded 985 individuals with missing values in the variables used to construct the polysocial score. The final analytic sample included 5000 participants, comprising 1848 men and 3152 women.

### Variables

#### Outcome

In the LSAHP, disability in each of the seven ADLs was assessed using the questions: 'Do you find it difficult to perform a task alone without the assistance of a person or assistive device due to your health or physical state?' Seven tasks were evaluated including taking a bath or shower, dressing, eating, standing up from a bed or chair, walking (around the house), going outside (leave the house), or using the toilet. Responses of "Yes" were taken to represent difficulty, and "Not sure" responses were marked as missing. Participants reporting difficulty in any ADL domain were classified as having ADL disability. Otherwise, they were classified as having no ADL disability.

#### Social determinants of health

**Fig. 1:**
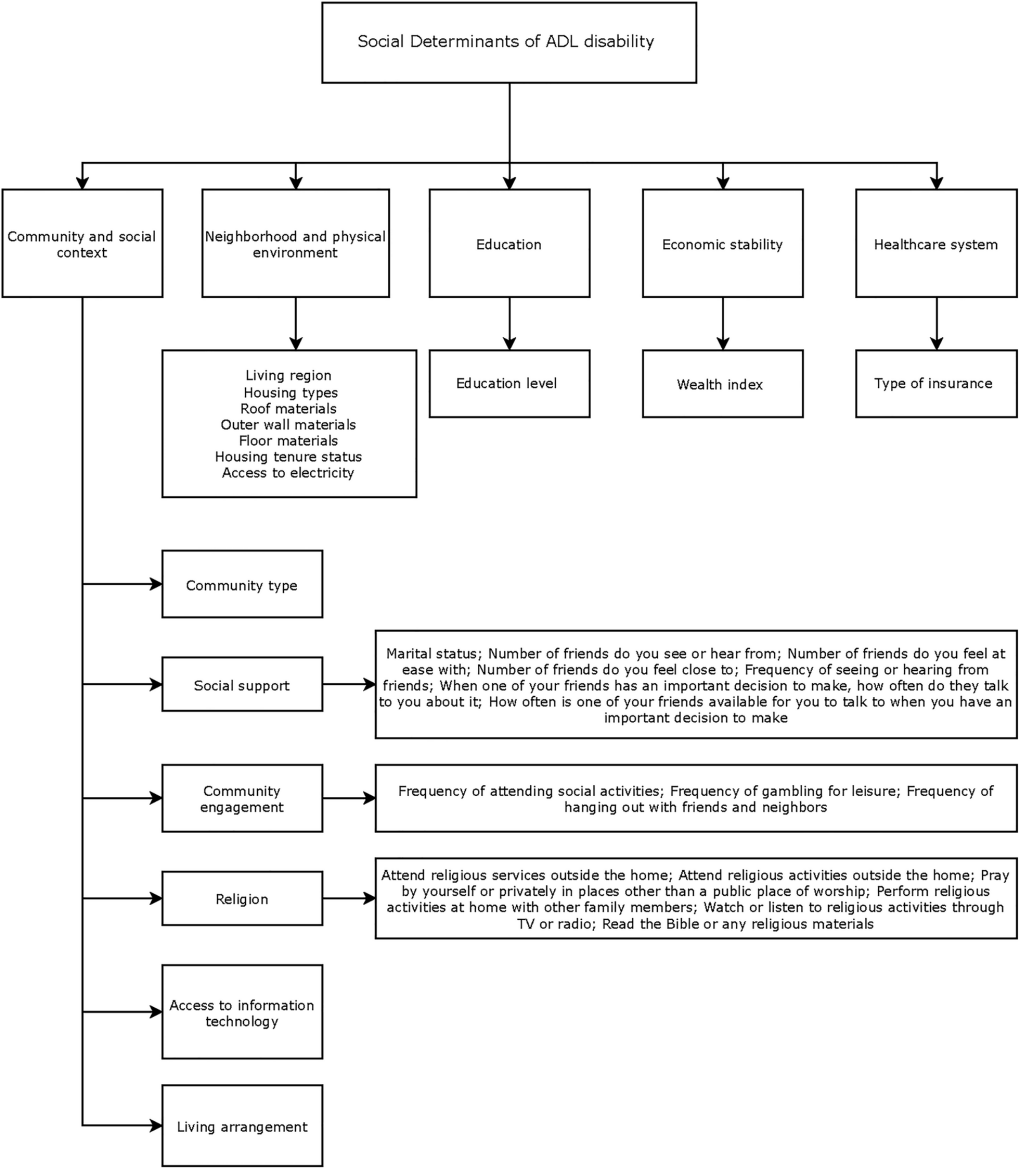
Social factors used for constructing polysocial score. The social determinants were grouped into five domains: community and social context, neighborhood and physical environment, education, economic stability, and the healthcare system. Each domain includes specific indicators such as social support, community engagement, living arrangement, and access to information technology

Based on prior research [22] and guided by the Healthy People 2030 Initiative framework (https://health.gov/healthypeople), which outlines five key domains of social determinants of health, namely economic stability, education access and quality, healthcare access and quality, neighborhood and built environment, and social and community context, we selected 29 social factors from these domains to comprehensively assess the individual-level social environment for both genders (Fig. 1 and Table S1).

**Economic stability:** Using information on the ownership of various assets, the wealth index quantifies the overall living standards of a household as a composite measure. It categorizes each household covered in the survey into quintiles from 1 (poorest) to 5 (wealthiest) [23].

**Neighborhood and physical environment:** The living region was categorized as urban or rural areas. Housing types were categorized as single houses or others. Roof materials were categorized as strong materials or lighter or mixed materials. Outer wall materials were categorized as strong materials or lighter or mixed materials. Floor materials were categorized as ceramic tiles, cement, or other materials. Housing tenure status was categorized as own house or rent house. Access to electricity was categorized as yes or no.

**Education:** Participants’ education was classified into three groups: below elementary, high school, and college or higher.

**Community and social context:** Community type was classified as city, población, or rural. The barangay is the smallest political unit in the Philippines. Each province or independent city comprises municipalities or component cities, further divided into barangays. Poblacion is the administrative center, which may take up the area of a single barangay or multiple barangays. Rural barangays have a low population density, with occupations focused on agriculture, fishing, and food gathering. Participants’ marital status was grouped as unmarried/separated, married, or widowed. Social support [24] refers to the presence of assistance available to an individual through their social relationships, including children, relatives, and friends. In our studies, we included variables like the number of friends you see or hear from, frequency of attending social activities, frequency of seeing or hearing from friends, living arrangement etc. Religion [25] measured participant’s religious attendance, engagement with religious media, and participation in private religious activities. Community engagements were measured by three aspects: frequency of attending social activities, frequency of gambling for leisure, and frequency of hanging out with friends and neighbors. Access to information technology (Information technology and aging) was categorized based on whether participants had access to the internet, a cell phone, a tablet, or a laptop. If participants had access to any of these, they were considered to have access to information technology.

**Healthcare system:** Healthcare insurance coverage was categorized based on whether the individual had any type of insurance (public or private).

#### Covariates

Demographic variables comprised age groups (60–69, 70–79, or 80 + years). Lifestyle factors included smoking status (never, former, or current), alcohol use frequency (no use, less than once a month, once a week to once or twice a month, and once every two or three days up to daily) and trouble when sleeping was classified as never, rarely, sometimes, and most of the time.

### Statistical analysis

Demographic and lifestyle variables were described for the overall sample and stratified by gender, with continuous variables presented as means and standard deviations (SD), and categorical variables as counts and percentages. Comparisons between genders employed the t test for continuous data and the Chi-square test for categorical data.

We used the light gradient boosting machine (LGBM) classifier [26] to select the important social factors for ADL disability among men and women, respectively. LGBM is an ensemble method that aggregates the predictions of multiple decision trees to make a final, well-generalized prediction. The Dropouts Meet Multiple Additive Regression Trees (DART) booster was employed during model training to increase accuracy and prevent overfitting [27, 28]. Factors were ranked based on their information gain, indicating their predictive value for ADL disability. LGBM classifiers were then sequentially constructed by adding variables based on their importance ranking. The iterative process ended once the highest AUC [29] was attained, determined by the absence of incremental gain in two consecutive DeLong statistical tests [30]. Then, we used logistic regression to determine the association between social factors selected from the LGBM classifier and ADL disability. Sampling weights were applied for all analyses in the study [21].

The polysocial score was developed separately by gender as a weighted sum of social factors, using raw regression coefficients as weights. To calculate each participant’s score, the scores of all individual social factors were summed, with each factor’s score determined by multiplying the absolute value of its regression coefficient by ten [14], where the reference category was assigned a score of 0. For example, among men, participants who attended social activities “a few times a year” were assigned 6 points (|–0.61|× 10), while those not attending social activities received 0 points (reference group). Similarly, for the variable attending religious services outside the home, individuals who answered “yes” received 3 points (|–0.26|× 10), whereas those answering “no” (reference group) received 0 points (Table S4). The final polysocial score was calculated by summing all such weighted values, reflecting the cumulative social environment at the individual level. The score was used as a continuous variable and categorized as low (quintile 1), intermediate (quintiles 2–4), and high (quintile 5) risk [31, 32], with cutoff points for men at 0–37, 38–63, and 64 + , and for women at 0–47, 48–69, and 70 + , respectively.

After creating the polysocial score among men and women, we used the locally weighted scatterplot smoothing (LOWESS), a nonparametric and robust curve fitting approach [33], to graphically inspect the shape of its relation with ADL disability. Logistic regression analyses were conducted to explore how polysocial scores, considered both as continuous measures and categorical groups, associated with ADL disability in both genders. Age, smoking status, current drinking frequency, and trouble when sleeping were included in the adjusted models. We will employ tenfold cross-validation to evaluate model performance (discrimination, calibration, and reclassification) among men and women participants. Model discrimination was measured using the C-statistic. For calibration, we applied the Hosmer–Lemeshow test and constructed a calibration plot to compare actual and estimated ADL disability risks within each decile of predicted probability. We used the net reclassification index (NRI) to assess reclassification; the reference model only included demographic (age) and lifestyle factors (smoking status, current drinking, and trouble when sleeping). To assess potential selection bias and the robustness of our findings, we conducted a sensitivity analysis using multiple imputation by chained equations (MICE) to impute missing values. Results obtained from imputed datasets were compared with those from the complete-case analysis to assess the robustness of variable selection.

All tests were two-sided with a significant level of 0.05. All statistical analyses were conducted in R 4.3.2 and Stata 18.0.

## Results

### Sample characteristics

Among the 5,000 individuals included in the study, the mean age was 68.3 years (SD = 6.8), and women accounted for 3,152 participants (63.0%; Table 1). Men were generally younger, more likely to smoke and consume alcohol, and reported fewer difficulties with sleep compared to women. Overall, 18.9% of participants were classified as having ADL disability, with a higher prevalence observed among women than men (20.3% vs. 16.9%).

**Table 1:** Descriptive characteristics of weighted sample

Characteristics	Total N = 5000	Men N = 1,848	Women N = 3,152	*P* value
Age in years (mean, SD)	68.3 (6.8)	67.9 (6.5)	68.5 (7.0)	0.12
Age group, years				
60–69	66.2%	70.0%	63.6%	0.06
70–79	25.8%	23.0%	27.8%	
80 +	8.0%	7.0%	8.6%	
Smoking status				
Current	17.4%	30.3%	8.5%	< 0.001
Former	25.9%	46.7%	11.5%	
Never	56.7%	23.0%	78.0%	
Current drinking				
No alcohol use	69.9%	48.8%	84.3%	< 0.001
Less than once a month	4.3%	10.0%	0.5%	
Once a week to once or twice a month	7.9%	15.6%	2.7%	
Once every two or three days to everyday	17.9%	25.6%	12.5%	
Trouble when sleeping				
Never	22.8%	28.3%	19.0%	0.01
Rarely	29.8%	30.7%	29.1%	
Sometimes	32.4%	28.9%	34.9%	
Most of the time	15.0%	12.1%	17.0%	
ADL disability				0.29
No	81.1%	83.1%	79.7%	
Yes	18.9%	16.9%	20.3%	

*P* values were obtained using the t test for continuous variables and the chi-squared test for categorical variables adjusting for sample weights

*SD* Standard deviation

### Creation of the polysocial score

**Fig. 2:**
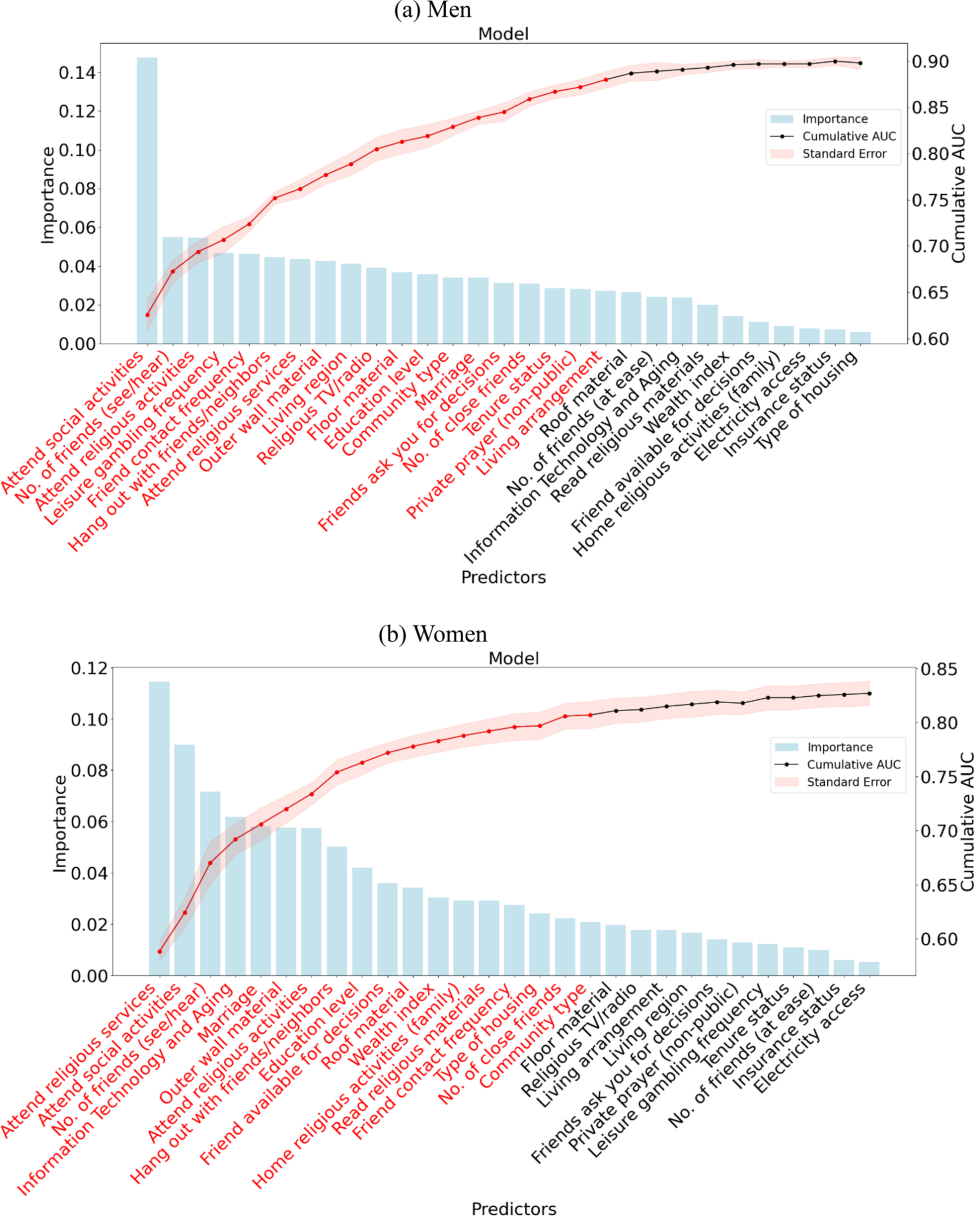
Importance ranking of social factors based on LGBM. The bar chart indicates the importance of the sorted proteins based on their contributions to the ADL disability prediction (as judged by the information gain). The line chart illustrates cumulative AUC values (right axis) upon including social determinant individually. Shaded regions represent standard errors derived from cross-validation. Predictor names are abbreviated for clarity. See Table S6 for full descriptions

We initially included 29 social factors in the LGBM; the distribution of the polysocial score was nearly normal among men and women (Figure S1); the final model retained 19 and 18 factors to develop the polysocial score for men and women, respectively (Fig. 2, and Tables S2 and S3). The top five social factors associated with ADL disability among men were frequency of attending social activities, number of friends they see or hear from, attending religious activities outside the home, frequency of gambling for leisure, and frequency of seeing or hearing from friends. The top five social factors among women were attending religious services outside the home, frequency of attending social activities, having a cell phone, number of friends you see or hear from, and marital status. We found 11 overlapping factors between men and women, such as attending religious services outside the home, frequency of attending social activities, and frequency of hanging out with friends and neighbors. Some variables were retained for men only, such as the frequency of gambling for leisure and the living arrangement of older persons, while others were kept for women only, such as the wealth index (Tables S4 and S5). Among men, the polysocial score spanned from 3 to 102, while among women, it ranged from 10 to 102, with higher scores denoting better social conditions. The mean value was 50.0 (SD = 14.7) among men and 58.1 (SD = 12.8) among women.

### Association between polysocial score and ADL disability

A monotonic and non-linear inverse association between polysocial score and ADL disability was observed in the LOWESS plot (Figure S2). Specifically, the majority of men with ADL disability were in the low score group (38.0%), followed by 12.7% in the intermediate and 4.0% in the high group. Similarly, 43.6% of women with ADL disability had low scores, compared to 18.1% and 6.3% in the intermediate and high score groups, respectively (Table 2).

**Table 2:** Association of polysocial score with ADL disability

Polysocial score	ADL disabled (%)	OR (95% CI)	
		Unadjusted	Adjusted^a^
Men (N = 1,848)			
Continuous		0.94 (0.92, 0.97)	0.94 (0.92, 0.96)
Categories			
Low: 0–37	38.0	Ref	Ref
Intermediate: 38–63	12.7	0.23 (0.12, 0.47)	0.22 (0.11, 0.41)
High: 64 +	4.0	0.07 (0.03, 016)	0.06 (0.02, 0.14)
Women (N = 3,152)			
Continuous		0.94 (0.92, 0.96)	0.94 (0.92, 0.96)
Categories			
Low: 0–47	43.6	Ref	Ref
Intermediate: 48–69	18.1	0.29 (0.16, 0.52)	0.28 (0.15, 0.52)
High: 70 +	6.3	0.09 (0.04, 0.18)	0.10 (0.04, 0.20)

^a^The adjusted models included age, smoking, drinking, and trouble when sleeping

*OR* Odds ratio; *CI* Confidence interval

After multivariable adjustment, men with intermediate and high polysocial scores had significantly lower odds of experiencing ADL disability—by 78% (95% CI: 59–89%) and 94% (95% CI: 86–98%), respectively—compared to those with low scores (Table 2). Similarly, women classified in the intermediate and high polysocial score categories experienced 72% and 90% lower odds of ADL disability than those in the low group. Treating the score continuously, each one-point increment was linked to a 6% (95% CI: 4–8%) reduction in ADL disability odds among both gender.

Good discrimination was observed in both continuous and categorical models of the polysocial score, with a significant increase in C-statistic of 0.06 and 0.05 for men, and 0.03 for women, respectively (Table 3). In both models for women, the Hosmer–Lemeshow statistics showed satisfactory calibration; the agreement between observed and predicted ADL disability was high (Figure S3). Models including the continuous and categorical polysocial score had an NRI of 0.37 and 0.18 for women, respectively. The prediction performance of both models for ADL disability showed good calibration and reclassification results among men.

**Table 3:** Performance metrics of prevalence of ADL disability for the continuous and categorical polysocial score

	Cohort	Reference model^a^	Reference + continuous polysocial score	Reference + categorical polysocial score
C-statistic	Men	0.66 (0.62, 0.70)	0.72 (0.69, 0.76)	0.71 (0.68,0.75)
	Women	0.70 (0.68, 0.72)	0.73 (0.71, 0.75)	0.73 (0.71,0.75)
∆ C-statistic	Men	Ref	0.06 (P < 0.001)	0.05 (P < 0.001)
	Women	Ref	0.03 (P < 0.001)	0.03 (P < 0.001)
Hosmer–Lemeshow statistic	Men	9.12 (P = 0.33)	24.6 (P = 0.002)	8.59 (P = 0.38)
	Women	9.78 (P = 0.28)	6.39 (P = 0.61)	11.23 (P = 0.19)
NRI	Men	Ref	0.52 (0.33, 0.80)	0.26 (0.15, 0.42)
	Women	Ref	0.37 (0.31, 0.56)	0.18 (0.13, 0.29)
Event NRI	Men	Ref	0.32 (-0.48, 0.57)	-0.01 (-0.14, 0.16)
	Women	Ref	0.23 (-0.35, 0.38)	-0.01 (-0.10, 0.12)
Non-event NRI	Men	Ref	0.20 (0.12, 0.85)	0.27 (0.07, 0.47)
	Women	Ref	0.14 (0.08, 0.69)	0.19 (0.10, 0.36)

^a^Reference model included age, smoking, drinking, and trouble when sleeping

*C-statistic* Concordance statistic, *NRI* Net reclassification index

### Sensitivity analysis

The multiple imputation analysis yielded results largely consistent with the complete-case analysis for variables selection. For men, 19 predictors were selected in the complete-case analysis and 18 in the imputed data, with 15 overlapping. For women, 18 predictors were selected in the complete-case analysis and 19 in the imputed data, with 16 overlapping. This high consistency suggests that missing data did not substantially affect variable selection or the robustness of the findings (Tables S7 and S8).

## Discussion

Drawing on data from a nationally representative survey, we developed and validated a polysocial score to estimate the risk of ADL disability in the aging population of the Philippines. The score encompassed 19 social determinants for men and 18 for women, spanning five key domains: economic stability, neighborhood and physical environment, education, community and social relationships, and the healthcare system. A higher polysocial score was associated with a reduced prevalence of ADL disability. Our analysis revealed gender differences in constructing the polysocial score, with different numbers and types of social factors selected for men and women.

We found 19 social factors in constructing the polysocial score among men; the top five were from two categories: social engagement (frequency of attending social activities, number of friends you see or hear from, frequency of gambling for leisure, and frequency of seeing or hearing from friends) and religion participation (attending religious activities outside the home). While constructing the polysocial score for women, there were 18 social factors, and the top five were also from two categories: social engagement (frequency of attending social activities, number of friends you see or hear from, information technology and aging, and marital status) and religion activities (attending religious services outside the home). The top five social factors shared by both men and women included attending religious services outside the home, frequency of attending social activities, and number of friends they see or hear from. Having friends and social participation were negatively associated with ADL disability prevalence [34]. Participation in religious services (at least once a week) was associated with a lower likelihood of experiencing ADL disability in both older men and women, compared to those with less frequent or no attendance [35]. However, these studies did not rank the importance of social factors on ADL disability. As in our research, these social engagement and religious participation factors emerged as the top important factors for both genders. Baseline social isolation significantly predicted the onset of ADL disability in older women [36]. A review suggested that information and communication technologies could effectively tackle social isolation among older adults [37]. This might explain why information technology usage was a particularly important social factor for older women.

To deepen the interpretation of our findings, several conceptual models could help explain the mechanisms through which social factors influence functional health in later life. The Activity Theory of Ageing (ATA) [38] and the Social Support Model (SSM) [39] provided complementary perspectives on how social engagement supports functional health in older adults. According to the ATA, continued engagement in meaningful social roles and activities will promote positive outcomes for older adults. The SSM further highlighted the protective effects of interpersonal relationships that provide emotional and instrumental support. These frameworks together helped explain our findings that frequent social engagement and strong interpersonal connections were key protective factors against ADL disability. The Socioemotional Selectivity Theory (SST) [40] further reinforced the mechanisms proposed by the ATA and the SSM. As people age, they increasingly prioritized emotionally meaningful relationships, which strengthened the benefits of active social participation and supportive ties.

Eleven social factors were overlapped among men and women, including the top overlapped important factors for both groups mentioned above. They were from social engagement (frequency of attending social activities, number of friends you see or hear from, number of friends you feel close to, frequency of hanging out with friends and neighbors and frequency of seeing or hearing from friends, community type, and marital status), religion participation (attending religious activities outside the home and attending religious services outside the house), neighborhood and physical environment (outer wall material), and education (educational attainment). Prior research indicated that older adults with active social engagement or ties had a lower risk of ADL disability [34, 41, 42]. Among Chinese older adults, greater social engagement was associated with decreased odds of functional disability [43]. Similar associations between social engagement and ADL disability have also been reported in some Southeast Asian countries, such as Malaysia [44] and Thailand [45]. While our study focused on social engagement, such as interactions with friends and participation in community activities, it is important to recognize that family engagement also plays a vital role in the lives of older adults in the Philippines. Most older Filipinos lived with their children or other family members and received substantial intergenerational support [46]. This strong familial context may interact with social relationships in shaping functional outcomes. Future research could benefit from examining both social and family engagement together to provide a more comprehensive understanding of aging in the Philippine setting. A higher level of religious involvement, assessed by church attendance, was related to a lower level of functional disability, and religion and spirituality were significant strategies for coping with disability and various health outcomes [47, 48, 49]. Southeast Asia is one of the most religiously diverse regions in the world and its influence may be especially pronounced in the Philippines, where approximately 80% of the household population identifies as Roman Catholic, while those with no religious affiliation account for less than 0.1% [50]. This deeply rooted religious culture could potentially reinforce the protective effects of religious involvement on functional disability. In addition, community type tended to be associated with ADL disability. Indeed, a study found that significant disparities in disability prevalence are evident across different levels of urbanization, with rural U.S. residents experiencing the highest rates of disability [51]. Being married was associated with lower odds of experiencing ADL disability relative to being unmarried [12]. Although there was no clear evidence between outer wall materials and ADL disability, some literature found that built environments were contributing to the presence of disability [52, 53]. We found educational attainment to be an essential factor for ADL disability. Previous studies found that higher-educated older adults had a lower likelihood of ADL disability compared to lower-educated older adults. Still, the impact of education on transitions in disability is minimal [54, 55]. Our results align with earlier research that identified multiple social factors linked to disability.

A decomposition analysis across multiple countries revealed that approximately 45% of the disability gap between men and women aged 50 and above was explained by differences in social determinants [20]. Similarly, we also identified some important social factors related to ADL disability that were unique to either men or women. The frequency of gambling for leisure was important only for men. Indeed, a subgroup analysis revealed that playing cards or mahjong was significantly associated with a lower risk of ADL disability among men in China [56]. Gambling could be a social activity, and men were more likely to gamble than women Field, which may help explain why gender differences existed for ADL disability. However, we acknowledged that gambling may not universally represent positive social engagement. Its interpretation could be culturally sensitive and context-dependent, as gambling may carry negative social or health connotations in some settings [57]. Therefore, this finding should be interpreted with caution, and further research is warranted to disentangle the social versus potentially harmful aspects of such activities. The wealth index was an important factor for women in our study. A multi-site longitudinal survey revealed that insufficient income was associated with late-life disability for women [58]. Older adults with higher income levels exhibited significantly lower rates of ADL disability than their lower-income counterparts [59]. However, older women were more likely to experience poverty and financial distress [60, 61]. Consequently, income inequality may exacerbate gender inequality and increase their disadvantages. Overall, disparities in disability between genders may reflect broader social gender inequalities, highlighting the need to understand how health, economic, and cultural factors affect gender-specific healthcare usage among older adults in the country [62].

Our study demonstrated that both men and women living in more advantageous social environments experienced a lower prevalence of ADL disability. Our findings were in line with earlier U.S.-based research demonstrating a negative association between polysocial scores and ADL disability among older adults [19]. The model performance was comparable to other studies employing similar approaches with different health outcomes [14, 15, 63]. Considering the complex interplay among social determinants of health, analyzing each factor independently may yield insufficient insight for informing effective interventions and ignore the complex mechanisms through which social conditions could generate disadvantages [14]. Developing a polysocial score offers a potential solution for identifying at-risk older adults, as it could help design patient-centered interventions.

This study has several strengths. Primarily, it incorporated 29 social determinants and systematically evaluated their cumulative effects on ADL disability across gender groups. This comprehensive approach offers distinct advantages over conventional methods that analyze social factors individually. This method allowed for a more comprehensive representation of how various social factors collectively influence individuals, reflecting the complex and interconnected structure of the social environment. Second, the polysocial score was constructed by weighting each social factor based on its effect on ADL disability. This approach allows for differential consideration of each factor’s impact, capturing their relative importance in influencing functional limitations. To date, this study represents the initial effort to examine how polysocial scores relate to ADL disability among older adults in developing countries. This work establishes a foundation for future research aimed at identifying social factors that may mitigate ADL disability and enhance resilience in aging populations facing functional decline.

We acknowledged several limitations. Firstly, we only included social factors from the baseline year, which prevented us from measuring changes in the polysocial score during the follow-up years. Further studies are needed to characterize changes in the polysocial score over time and assess their impact on ADL disability. Second, ADL disability is not static but rather a dynamic process marked by transitions across multiple disability states [64]. Future investigations should focus on how social factors and polysocial scores impact the recovery from disability. Finally, while our study successfully constructed a polysocial score and identified significant social factors associated with ADL disability, it is important to acknowledge the possibility of reverse causality. The cross-sectional design of the LSAHP limits our ability to determine the causal direction between social factors and difficulties in ADL. For instance, instead of social disengagement leading to disability, it is also plausible that individuals with ADL disability reduce their social participation due to mobility limitations or dependence on others. Future studies employing longitudinal data would be essential to disentangle these relationships and better understand their directionality and causality.

## Conclusions

In conclusion, our study developed gender-specific polysocial scores reflecting multiple social determinants, including economic stability, neighborhood and physical environment, education, community and social context, and healthcare system. The demonstrated association between these scores and ADL disability highlights their strong reliability and validity as predictors of functional impairment risk among older Filipinos. The findings suggested the development of more precise social intervention to foster independence in older adults. However, due to the cross-sectional design, causal inference is limited, and reverse causality cannot be ruled out.

## Supplementary Information

Additional file 1.

## Data Availability

Data used in this study is available by applying to Demographic Research and Development Foundation website (https://www.drdf.org.ph/longitudinal-study-of-ageing-and-health-in-the-philippines/).

## Abbreviations

ADL: Activities of daily living
LightGBM: Light gradient boosting machine
LSAHP: Longitudinal study of ageing and health in the philippines
LOWESS: Locally weighted scatterplot smoothing
SD: Standard deviations
NRI: Net reclassification index
