# Supplementary material for: Exploring social determinants of disability among older filipinos: insights from a polysocial score approach
Source: Glob Health Res Policy. 2025 Oct 29;10:55. doi: 10.1186/s41256-025-00453-7 (PMC12570412; doi:10.1186/s41256-025-00453-7)
Supplement: Supplementary file 1 — Additional file 1. [file 41256_2025_453_MOESM1_ESM.docx]

**Supplementary materials**

- **Table S1.** Twenty-nine social factors selected from the Longitudinal Study of Ageing and Health in the Philippines.
- **Table S2.** Frequency of important social factors selected by LGBM among men.
- **Table S3.** Frequency of important social factors selected by LGBM among women.
- **Table S4.** LightGBM for selecting social variables for constructing the polysocial scores among men.
- **Table S5.** LightGBM for selecting social variables for constructing the polysocial scores among women.
- **Table S6.** Abbreviations of predictor variables used in the Figure 2.
- **Table S7.** Comparison of predictors between complete case and multiple imputation analyses in men.
- **Table S8.** Comparison of predictors between complete case and multiple imputation analyses in women.
- **Figure S1.** The distribution of the polysocial score.
- **Figure S2.** LOWESS plot graphs displaying incidence of ADLs by polysocial score among men and women.
- **Figure S3.** Agreement between observed and predicted prevalence of ADL disability for the continuous and categorical polysocial score.

**Table S1.** Twenty-nine social factors selected from the Longitudinal Study of Ageing and Health in the Philippines.

| Social factors | Men (N=1,848) | | Women (N=3,152) | | *P* value |
| --- | --- | --- | --- | --- | --- |
|  | Observation (N) | Percent (%) | Observation (N) | Percent (%) |  |
| **Economic stability** | | | | | |
| **Wealth index** |  |  |  |  |  |
| 1 (the poorest 20%) | 411 | 22.24 | 591 | 18.75 | <0.05 |
| 2 | 442 | 23.92 | 744 | 23.6 |  |
| 3 | 359 | 19.43 | 680 | 21.57 |  |
| 4 | 344 | 18.61 | 642 | 20.37 |  |
| 5 (the richest 20%) | 292 | 15.8 | 495 | 15.7 |  |
| **Community and social context** | | | | | |
| **Community type** |  |  |  |  |  |
| City | 176 | 9.52 | 314 | 9.96 | 0.658 |
| Poblacion | 282 | 15.26 | 504 | 15.99 |  |
| Rural | 1,390 | 75.22 | 2,334 | 74.05 |  |
| **Marital status** |  |  |  |  |  |
| Currently unmarried | 279 | 15.1 | 371 | 11.77 | <0.01 |
| Currently married | 1,116 | 60.39 | 817 | 25.92 |  |
| Widowed | 453 | 24.51 | 1,964 | 62.31 |  |
| **Number of friends do you see or hear from** |  |  |  |  |  |
| Less than | 336 | 18.18 | 716 | 22.72 | <0.01 |
| 3-4 | 261 | 14.12 | 541 | 17.16 |  |
| 5-8 | 279 | 15.1 | 461 | 14.63 |  |
| 9+ | 972 | 52.6 | 1,434 | 45.49 |  |
| **Number of friends do you feel at ease with** |  |  |  |  |  |
| 0 | 406 | 21.97 | 703 | 22.3 | <0.01 |
| 1 | 332 | 17.97 | 783 | 24.84 |  |
| 2 | 405 | 21.92 | 715 | 22.68 |  |
| 3-4 | 325 | 17.59 | 518 | 16.43 |  |
| 5+ | 380 | 20.56 | 433 | 13.74 |  |
| **Number of friends do you feel close to** |  |  |  |  |  |
| 0 | 370 | 20.02 | 653 | 20.72 | <0.01 |
| 1 | 379 | 20.51 | 894 | 28.36 |  |
| 2 | 436 | 23.59 | 782 | 24.81 |  |
| 3-4 | 312 | 16.88 | 475 | 15.07 |  |
| 5+ | 351 | 18.99 | 348 | 11.04 |  |
| **Frequency of seeing or hearing from friends** |  |  |  |  |  |
| Never or seldom | 745 | 40.31 | 1,258 | 39.91 | = 0.896 |
| Sometimes | 511 | 27.65 | 864 | 27.41 |  |
| (Very) Often or always | 592 | 32.03 | 1,030 | 32.68 |  |
| **When one of your friends has an important decision to make, how often do they talk to you about it** |  |  |  |  |  |
| Never | 259 | 14.02 | 444 | 14.09 | = 0.312 |
| Seldom | 776 | 41.99 | 1,242 | 39.4 |  |
| Sometimes | 520 | 28.14 | 934 | 29.63 |  |
| (Very) Often or always | 293 | 15.85 | 532 | 16.88 |  |
| **How often is one of your friends available for you to talk to when you have an important decision to make?** |  |  |  |  |  |
| Never | 269 | 14.56 | 470 | 14.91 | = 0.155 |
| Seldom | 826 | 44.7 | 1,307 | 41.47 |  |
| Sometimes | 484 | 26.19 | 889 | 28.2 |  |
| (Very) Often or always | 269 | 14.56 | 486 | 15.42 |  |
| **Frequency of attending social activities** |  |  |  |  |  |
| Never | 697 | 37.72 | 1,197 | 37.98 | <0.01 |
| A few times a year | 621 | 33.6 | 941 | 29.85 |  |
| About once a month or more often | 530 | 28.68 | 1,014 | 32.17 |  |
| **Frequency of gambling for leisure** |  |  |  |  |  |
| Never | 1,582 | 85.61 | 2,961 | 93.94 | <0.01 |
| A few times a year or more often | 266 | 14.39 | 191 | 6.06 |  |
| **Frequency of hanging out with friends and neighbors** |  |  |  |  |  |
| Never or a few times a year | 594 | 32.14 | 1,145 | 36.33 | <0.05 |
| About once a month or more often | 419 | 22.67 | 668 | 21.19 |  |
| Several times a week | 482 | 26.08 | 746 | 23.67 |  |
| Everyday | 353 | 19.1 | 593 | 18.81 |  |
| **Attend religious services outside the home** |  |  |  |  |  |
| Yes | 1,244 | 67.32 | 2,572 | 81.6 | <0.01 |
| No | 604 | 32.68 | 580 | 18.4 |  |
| **Attend religious activities outside the home** |  |  |  |  |  |
| Yes | 448 | 24.24 | 1,154 | 36.61 | <0.01 |
| No | 1,400 | 75.76 | 1,998 | 63.39 |  |
| **Pray by yourself or privately in places other than a public place of worship** |  |  |  |  |  |
| Yes | 1,050 | 56.82 | 2,221 | 70.46 | <0.01 |
| No | 798 | 43.18 | 931 | 29.54 |  |
| **Perform religious activities at home with other family members** |  |  |  |  |  |
| Yes | 390 | 21.1 | 1,060 | 33.63 | <0.01 |
| No | 1,458 | 78.9 | 2,092 | 66.37 |  |
| **Watch or listen to religious activities through TV or radio** |  |  |  |  |  |
| Yes | 1,128 | 35.79 | 1,128 | 35.79 | = 0.171 |
| No | 2,024 | 64.21 | 2,024 | 64.21 |  |
| **Read the Bible or any religious materials** |  |  |  |  |  |
| Yes | 460 | 24.89 | 1,135 | 36.01 | <0.01 |
| No | 1,388 | 75.11 | 2,017 | 63.99 |  |
| **Information Technology and Aging (yes in any)** |  |  |  |  |  |
| No | 1,321 | 71.48 | 2,127 | 67.48 | <0.05 |
| Yes in any | 527 | 28.52 | 1,025 | 32.52 |  |
| **Living arrangement** |  |  |  |  |  |
| Living alone | 212 | 11.47 | 444 | 14.09 | <0.01 |
| Living with spouse only | 245 | 13.26 | 235 | 7.46 |  |
| Living with children | 1,119 | 60.55 | 1,852 | 58.76 |  |
| Other types of arrangement | 272 | 14.72 | 621 | 19.7 |  |
| **Neighborhood and physical environment** | | | | | |
| **Living region** |  |  |  |  |  |
| Urban | 737 | 39.88 | 1,440 | 45.69 | <0.01 |
| Rural | 1,111 | 60.12 | 1,712 | 54.31 |  |
| **Type of housing** |  |  |  |  |  |
| Single house | 1,773 | 95.94 | 2,994 | 94.99 | = 0.122 |
| Other | 75 | 4.06 | 158 | 5.01 |  |
| **Roof material** |  |  |  |  |  |
| Strong materials | 1,544 | 84 | 2,631 | 83 | = 0.942 |
| Lighter or mixed materials | 304 | 16.45 | 521 | 16.53 |  |
| **Outer wall materials** |  |  |  |  |  |
| Strong material | 1,003 | 54.27 | 1,814 | 57.55 | = 0.077 |
| Lighter material | 231 | 12.5 | 371 | 11.77 |  |
| Other material | 614 | 33.23 | 967 | 30.68 |  |
| **Floor material** |  |  |  |  |  |
| Ceramic tiles | 371 | 20.08 | 649 | 20.59 | = 0.014 |
| Cement | 976 | 52.81 | 1,764 | 55.96 |  |
| Other material | 501 | 27.11 | 739 | 23.45 |  |
| **Tenure status** |  |  |  |  |  |
| Own house and lot or owner-like possession of house and lot | 1,234 | 66.77 | 2,088 | 66.24 | = 0.701 |
| Rent house or lot or both, either rent-free or not, with or without owner's consent | 614 | 33 | 1,064 | 34 |  |
| **Access to electricity** |  |  |  |  |  |
| Yes | 1,671 | 90 | 2,957 | 94 | <0.01 |
| No | 177 | 10 | 195 | 6 |  |
| **Healthcare system** | | | | | |
| **With any insurance (public or private)** |  |  |  |  |  |
| Yes | 1,426 | 77 | 2,436 | 77 | = 0.922 |
| No | 422 | 23 | 716 | 23 |  |
| **Education** | | | | | |
| **Educational attainment** |  |  |  |  |  |
| Elementary level or lower | 1,167 | 63 | 2,101 | 66.66 | <0.05 |
| High school level | 493 | 27 | 739 | 23.45 |  |
| College level or higher | 188 | 10.17 | 312 | 10 |  |

**Table S2.** Frequency of 19 important social factors selected by LGBM in men.

|  | Men (N=1,848) | |
| --- | --- | --- |
| Order | Social factors | Frequency (Percentage) |
| **1** | **Frequency of attending social activities** |  |
|  | Never | 697 (37.72) |
|  | A few times a year | 621 (33.6) |
|  | About once a month or more often | 530 (28.68) |
| **2** | **No. of friends do you see or hear from** |  |
|  | Less than 3 | 336 (18.18) |
|  | 3-4 | 261 (14.12) |
|  | 5-8 | 279 (15.1) |
|  | 9+ | 972 (52.6) |
| **3** | **Attend religious activities outside the home** |  |
|  | Yes | 448 (24.24) |
|  | No | 1.400 (75.76) |
| **4** | **Frequency of gambling for leisure** |  |
|  | Never | 1,582 (85.61) |
|  | A few times a year or more often | 266 (14.39) |
| **5** | **Frequency of seeing or hearing from friends** |  |
|  | Less often | 745 (40.31) |
|  | Sometimes | 511 (27.65) |
|  | More often | 592 (32.03) |
| **6** | **Frequency of hanging out with friends and neighbors** |  |
|  | Never or a few times a year | 594 (32.14) |
|  | About once a month or more often | 419 (22.67) |
|  | Several times a week | 482 (26.08) |
|  | Everyday | 353 (19.1) |
| **7** | **Attend religious services outside the home** |  |
|  | Yes | 1,244 (67.32) |
|  | No | 604 (32.68) |
| **8** | **Outer wall materials** |  |
|  | Strong material | 1,003 (54.27) |
|  | Lighter material | 231 (12.50) |
|  | Other material | 614 (33.23) |
| **9** | **Living region** |  |
|  | Rural | 1,111 (60.12) |
|  | Urban | 737 (39.88) |
| **10** | **Watch or listen to religious activities through TV or radio** |  |
|  | Yes | 626 (33.87) |
|  | No | 1,222 (66.13) |
| **11** | **Floor material** |  |
|  | Ceramic tiles | 371 (20.08) |
|  | Cement | 976 (52.81) |
|  | Other material | 501 (27.11) |
| **12** | **Educational attainment** |  |
|  | Elementary level or lower | 1,167 (63.15) |
|  | High school level | 493 (26.68) |
|  | College level or higher | 188 (10.17) |
| **13** | **Community type** |  |
|  | City | 176 (9.52) |
|  | Poblacion | 282 (15.26) |
|  | Rural | 1390 (75.22) |
| **14** | **Marriage** |  |
|  | Currently unmarried/separated | 279 (15.1) |
|  | Currently married | 1,116 (60.39) |
|  | Widowed | 453 (24.51) |
| **15** | **When one of your friends has an important decision to make, how often do they talk to you about it** |  |
|  | Never | 259 (14.02) |
|  | Seldom | 776 (41.99) |
|  | Sometimes | 520 (28.14) |
|  | Often or more | 293 (15.85) |
| **16** | **No. of friends do you feel close to** |  |
|  | 0 | 370 (20.02) |
|  | 1 | 379 (20.51) |
|  | 2 | 436 (23.59) |
|  | 3-4 | 312 (16.88) |
|  | 5+ | 351 (18.99) |
| **17** | **Tenure status** |  |
|  | Own house and lot | 1,234 (66.77) |
|  | Rent house or lot or both | 614 (33.23) |
| **18** | **Pray by yourself or privately in places other than a public place of worship** |  |
|  | Yes | 1,050 (56.82) |
|  | No | 798 (43.18) |
| **19** | **Living Arrangement of Older Person** |  |
|  | Living alone | 212 (11.47) |
|  | Living with spouse only | 245 (13.26) |
|  | Living with children | 1,119 (60.55) |
|  | Other types of arrangement | 272 (14.72) |

Note:

1. The 'Order' column represents the importance ranking of the variables.

**Table S3.** Frequency of 18 important social factors selected by LGBM in women.

|  | Women (N=3,152) | |
| --- | --- | --- |
| Order | Social factors | Frequency (Percentage) |
| **1** | **Attend religious services outside the home** |  |
|  | Yes | 2,572 (81.6) |
|  | No | 580 (18.4) |
| **2** | **Frequency of attending social activities** |  |
|  | Never | 1,197 (37.98) |
|  | A few times a year | 941 (29.85) |
|  | About once a month or more often | 1,014 (32.17) |
| **3** | **No. of friends do you see or hear from** |  |
|  | Less than 3 | 716 (22.72) |
|  | 3-4 | 541 (17.16) |
|  | 5-8 | 461 (14.63) |
|  | 9+ | 1,434 (45.49) |
| **4** | **Information technology and aging** |  |
|  | No | 2,127 (67.48) |
|  | Yes in any | 1,025 (32.52) |
| **5** | **Marital status** |  |
|  | Currently unmarried/separated | 371 (11.77) |
|  | Currently married | 817 (25.92) |
|  | Widowed | 1,964 (62.31) |
| **6** | **Outer wall material** |  |
|  | Strong material | 1,814 (57.55) |
|  | Lighter material | 371(11.77) |
|  | Other material | 967 (30.68) |
| **7** | **Attend religious activities outside the home** |  |
|  | Yes | 1,154 (36.61) |
|  | No | 1,998 (63.39) |
| **8** | **Frequency of hanging out with friends and neighbors** |  |
|  | Never or a few times a year | 1,145 (36.33) |
|  | About once a month or more often | 668 (21.19) |
|  | Several times a week | 746 (23.67) |
|  | Everyday | 593 (18.81) |
| **9** | **Educational attainment** |  |
|  | Elementary level or lower | 2,101 (66.66) |
|  | High school level | 739 (23.45) |
|  | College level or higher | 312 (9.9) |
| **10** | **How often is one of your friends available for you to talk to when you have an important decision to make?** |  |
|  | Never | 470 (14.91) |
|  | Seldom | 1,307 (41.47) |
|  | Sometimes | 889 (28.2) |
|  | Often or more | 486 (15.42) |
| **11** | **Roof material** |  |
|  | Strong materials | 2,631 (83.47) |
|  | Lighter or mixed materials | 521 (16.53) |
| **12** | **Wealth index** |  |
|  | Lowest | 591 (18.75) |
|  | Second | 744 (23.6) |
|  | Middle | 680 (21.57) |
|  | Fourth | 642 (20.37) |
|  | Highest | 495 (15.7) |
| **13** | **Perform religious activities at home with other family members** |  |
|  | Yes | 1,060 (33.63) |
|  | No | 2,092 (66.37) |
| **14** | **Read the Bible or any religious materials** |  |
|  | Yes | 1,135 (36.01) |
|  | No | 2,017 (63.99) |
|  |  |  |
| **15** | **How often do you see or hear from friends?** |  |
|  | Less often | 1,258 (39.91) |
|  | Sometimes | 864 (27.41) |
|  | More often | 1,030 (32.68) |
| **16** | **Type of housing** |  |
|  | Single house | 2,994 (94.99) |
|  | Other | 158 (5.01) |
| **17** | **No. of friends do you feel close to** |  |
|  | 0 | 653 (20.72) |
|  | 1 | 894 (28.36) |
|  | 2 | 782 (24.81) |
|  | 3-4 | 475 (15.07) |
|  | 5+ | 348 (11.04) |
| **18** | **Community type** |  |
|  | City | 314 (9.96) |
|  | Poblacion | 504 (15.99) |
|  | Rural | 2,334 (74.05) |

Note:

1. The 'Order' column represents the importance ranking of the variables.

**Table S4.** LightGBM for selecting social variables for constructing the polysocial scores among men.

|  | Men (N=1,848) | | |
| --- | --- | --- | --- |
| Order | Social factors | Coefficients | Score |
| **1** | **Frequency of attending social activities** |  |  |
|  | Never | Ref. | 0 |
|  | A few times a year | -0.61 | 6 |
|  | About once a month or more often | -0.18 | 2 |
| **2** | **No. of friends do you see or hear from** |  |  |
|  | 5-8 | Ref. | 0 |
|  | Less than 3 | -0.39 | 4 |
|  | 3-4 | -0.45 | 5 |
|  | 9+ | -0.60 | 6 |
| **3** | **Attend religious activities outside the home** |  |  |
|  | No | Ref. | 0 |
|  | Yes | -1.31 | 13 |
| **4** | **Frequency of gambling for leisure** |  |  |
|  | Never | Ref. | 0 |
|  | A few times a year or more often | -1.01 | 10 |
| **5** | **How often do you see or hear from friends** |  |  |
|  | More often | Ref. | 0 |
|  | Sometimes | -0.61 | 6 |
|  | Less often | -0.83 | 8 |
| **6** | **Frequency of hanging out with friends and neighbors** |  |  |
|  | Never or a few times a year | Ref. | 0 |
|  | About once a month or more often | -0.37 | 4 |
|  | Several times a week | -0.40 | 4 |
|  | Everyday | -0.51 | 5 |
| **7** | **Attend religious services outside the home** |  |  |
|  | No | Ref. | 0 |
|  | Yes | -0.26 | 3 |
| **8** | **Outer wall materials** |  |  |
|  | Other material | Ref. | 0 |
|  | Strong material | -0.22 | 2 |
|  | Lighter material | -0.38 | 4 |
| **9** | **Living region** |  |  |
|  | Urban | Ref. | 0 |
|  | Rural | -0.36 | 4 |
| **10** | **Watch or listen to religious activities through TV or radio** |  |  |
|  | Yes | Ref. | 0 |
|  | No | -0.55 | 6 |
| **11** | **Floor material** |  |  |
|  | Ceramic tiles | Ref. | 0 |
|  | Cement | -0.22 | 2 |
|  | Other material | -0.08 | 1 |
| **12** | **Educational attainment** |  |  |
|  | High school level | Ref. | 0 |
|  | Elementary level or lower | -0.16 | 2 |
|  | College level or higher | -0.63 | 6 |
| **13** | **Community type** |  |  |
|  | Rural |  |  |
|  | City | -1.83 | 18 |
|  | Poblacion | -0.55 | 6 |
| **14** | **Marriage** |  |  |
|  | Currently unmarried/separated | Ref. | 0 |
|  | Currently married | -0.28 | 3 |
|  | Widowed | -0.49 | 5 |
| **15** | **When one of your friends has an important decision to make, how often do they talk to you about it** |  |  |
|  | Seldom | Ref. | 0 |
|  | Never | -0.14 | 1 |
|  | Sometimes | 0.00 | 0 |
|  | Often or more | -0.37 | 4 |
| **16** | **No. of friends do you feel close to** |  |  |
|  | 3-4 | Ref. | 0 |
|  | 0 | -1.00 | 10 |
|  | 1 | -0.15 | 2 |
|  | 2 | -0.84 | 8 |
|  | 5+ | -0.36 | 4 |
| **17** | **Tenure status** |  |  |
|  | Rent house or lot or both | Ref. | 0 |
|  | Own house and lot | -0.41 | 4 |
| **18** | **Pray by yourself or privately in places other than a public place of worship** |  |  |
|  | Yes | Ref. | 0 |
|  | No | -0.40 | 4 |
| **19** | **Living Arrangement of Older Person** |  |  |
|  | Living with children | Ref. | 0 |
|  | Living alone | -0.90 | 9 |
|  | Living with spouse only | -1.30 | 13 |
|  | Other types of arrangement | -0.79 | 8 |

Notes:

1. The coefficients were attained based on logistic regression.
2. Model was adjusted for age, smoking, drinking status and trouble when sleeping.

**Table S5.** LightGBM for selecting social variables for constructing the polysocial scores among women.

|  | Women (N=3,152) | | |
| --- | --- | --- | --- |
| Order | Social factors | Coefficients | Score |
| **1** | **Attend religious services outside the home** |  |  |
|  | No | Ref. | 0 |
|  | Yes | -0.89 | 9 |
| **2** | **Frequency of attending social activities** |  |  |
|  | A few times a year | Ref. | 0 |
|  | Never | -0.23 | 2 |
|  | About once a month or more often | -0.84 | 8 |
| **3** | **No. of friends do you see or hear from** |  |  |
|  | Less than 3 | Ref. | 0 |
|  | 3-4 | -0.86 | 9 |
|  | 5-8 | -0.27 | 3 |
|  | 9+ | -1.01 | 10 |
| **4** | **Information technology and aging** |  |  |
|  | No | Ref. | 0 |
|  | Yes in any | -0.66 | 7 |
| **5** | **Marital status** |  |  |
|  | Currently married | Ref. | 0 |
|  | Widowed | -0.53 | 5 |
|  | Currently unmarried/separated | -0.67 | 7 |
| **6** | **Outer wall material** |  |  |
|  | Strong material | Ref. | 0 |
|  | Lighter material | -0.80 | 8 |
|  | Other material | -1.01 | 10 |
| **7** | **Attend religious activities outside the home** |  |  |
|  | No | Ref. | 0 |
|  | Yes | -0.01 | 0 |
| **8** | **Frequency of hanging out with friends and neighbors** |  |  |
|  | Never or a few times a year | Ref. | 0 |
|  | About once a month or more often | -0.03 | 0 |
|  | Several times a week | -0.94 | 9 |
|  | Everyday | -1.02 | 10 |
| **9** | **Educational attainment** |  |  |
|  | Elementary level or lower | Ref. | 0 |
|  | High school level | -0.87 | 9 |
|  | College level or higher | -0.83 | 8 |
| **10** | **How often is one of your friends available for you to talk to when you have an important decision to make?** |  |  |
|  | Never | Ref. | 0 |
|  | Seldom | -0.57 | 6 |
|  | Sometimes | -0.64 | 6 |
|  | Often or more | -0.87 | 9 |
| **11** | **Roof material** |  |  |
|  | Strong materials | Ref. | 0 |
|  | Lighter or mixed materials | -0.31 | 3 |
| **12** | **Wealth index** |  |  |
|  | Middle | Ref. | 0 |
|  | Lowest | -0.37 | 4 |
|  | Second | -0.31 | 3 |
|  | Fourth | -0.44 | 4 |
|  | Highest | -0.24 | 2 |
| **13** | **Perform religious activities at home with other family members** |  |  |
|  | Yes | Ref. | 0 |
|  | No | -0.16 | 2 |
| **14** | **Read the Bible or any religious materials** |  |  |
|  | Yes | Ref. | 0 |
|  | No | -0.13 | 1 |
| **15** | **How often do you see or hear from friends?** |  |  |
|  | Sometimes | Ref. | 0 |
|  | Less often | -0.83 | 8 |
|  | More often | -0.32 | 3 |
| **16** | **Type of housing** |  |  |
|  | Single house | Ref. | 0 |
|  | Other | -1.17 | 12 |
| **17** | **No. of friends do you feel close to** |  |  |
|  | 2 | Ref. | 0 |
|  | 0 | -0.69 | 7 |
|  | 1 | -0.55 | 6 |
|  | 3-4 | -0.32 | 3 |
|  | 5+ | -0.64 | 6 |
| **18** | **Community type** |  |  |
|  | City | Ref. | 0 |
|  | Rural | -0.53 | 5 |
|  | Poblacion | -0.80 | 8 |

Notes:

1. The coefficients were attained based on logistic regression.
2. Model was adjusted for age, smoking, drinking status and trouble when sleeping.

**Table S6.** Abbreviations of predictor variables used in the Figure 2.

| **Original Name** | **Abbreviation** |
| --- | --- |
| Frequency of attending social activities | Attend social activities |
| No. of friends do you see or hear from | No. of friends (see/hear) |
| Attend religious activities outside the home | Attend religious activities |
| Frequency of gambling for leisure | Leisure gambling frequency |
| How often do you see or hear from friends | Friend contact frequency |
| Frequency of hanging out with friends and neighbors | Hang out w/ friends/neighbors |
| Attend religious services outside the home | Attend religious services |
| Outer wall material | Outer wall material |
| Living region | Living region |
| Watch or listen to religious activities through TV or radio | Religious TV/radio |
| Floor material | Floor material |
| Educational attainment | Education level |
| Community type | Community type |
| Marriage | Marriage |
| When one of your friends has an important decision to make, how often do they talk to you about it | Friends ask you for decisions |
| No. of friends do you feel close to | No. of close friends |
| Tenure status | Tenure status |
| Pray by yourself or privately in places other than a public place of worship | Private prayer (non-public) |
| Living Arrangement of Older Person | Living arrangement |
| Roof material | Roof material |
| No. of friends do you feel at ease with | No. of friends (at ease) |
| Information Technology and Aging | IT and aging |
| Read the Bible or any religious materials | Read religious materials |
| Wealth index | Wealth index |
| How often is one of your friends available for you to talk to when you have an important decision to make | Friend available for decisions |
| Perform religious activities at home with other family members | Home religious activities (family) |
| Access to electricity | Electricity access |
| With/without insurance | Insurance status |
| Type of housing | Type of housing |

**Table S7.** Comparison of predictors between complete case and multiple imputation analyses in men.

| **Complete case** | **Total Gain** | **Multiple imputation** | **Total**  **Gain** |
| --- | --- | --- | --- |
| **Frequency of attending social activities** | 0.15 | **Frequency of attending social activities** | 0.19 |
| **No. of friends do you see or hear from** | 0.06 | **Attend religious services outside the home** | 0.09 |
| **Attend religious activities outside the home** | 0.05 | **Frequency of hanging out with friends and neighbors** | 0.09 |
| **Frequency of gambling for leisure** | 0.05 | **No. of friends do you see or hear from** | 0.06 |
| **How often do you see or hear from friends** | 0.05 | **Attend religious activities outside the home** | 0.05 |
| **Frequency of hanging out with friends and neighbors** | 0.04 | **Frequency of gambling for leisure** | 0.04 |
| **Attend religious services outside the home** | 0.04 | **How often do you see or hear from friends** | 0.04 |
| **Outer wall material** | 0.04 | **No. of friends do you feel at ease with** | 0.03 |
| **Living region** | 0.04 | **Living region** | 0.03 |
| **Watch or listen to religious activities through TV or radio** | 0.04 | **Outer wall material** | 0.03 |
| **Floor material** | 0.04 | **Information Technology and Aging** | 0.03 |
| **Educational attainment** | 0.04 | **No. of friends do you feel close to** | 0.02 |
| **Community type** | 0.03 | **Community type** | 0.02 |
| **Marriage** | 0.03 | **Marriage** | 0.02 |
| **When one of your friends has an important decision to make, how often do they talk to you about it** | 0.03 | **When one of your friends has an important decision to make, how often do they talk to you about it** | 0.02 |
| **No. of friends do you feel close to** | 0.03 | **Tenure status** | 0.02 |
| **Tenure status** | 0.03 | **Educational attainment** | 0.02 |
| **Pray by yourself or privately in places other than a public place of worship** | 0.03 | **Wealth index** | 0.02 |
| **Living Arrangement of Older Person** | 0.03 | Roof material | 0.02 |
| Roof material | 0.03 | How often is one of your friends available for you to talk to when you have an important decision to make | 0.02 |
| No. of friends do you feel at ease with | 0.02 | Floor material | 0.02 |
| Information Technology and Aging | 0.02 | Living Arrangement of Older Person | 0.02 |
| Read the Bible or any religious materials | 0.02 | Access to electricity | 0.02 |
| Wealth index | 0.01 | Watch or listen to religious activities through TV or radio | 0.01 |
| How often is one of your friends available for you to talk to when you have an important decision to make | 0.01 | Read the Bible or any religious materials | 0.01 |
| Perform religious activities at home with other family members | 0.01 | Pray by yourself or privately in places other than a public place of worship | 0.01 |
| Access to electricity | 0.01 | With/without insurance | 0.01 |
| With/without insurance | 0.01 | Perform religious activities at home with other family members | 0.01 |
| type of housing | 0.01 | type of housing | 0.00 |

Note: Bold variables are selected by DeLong test.

**Table S8.** Comparison of predictors between complete case and multiple imputation analyses in women.

| **Complete case** | **Total Gain** | **Multiple imputation** | **Total**  **Gain** |
| --- | --- | --- | --- |
| **Attend religious services outside the home** | 0.11 | **Attend religious services outside the home** | 0.24 |
| **Frequency of attending social activities** | 0.09 | **Frequency of attending social activities** | 0.11 |
| **No. of friends do you see or hear from** | 0.07 | **Attend religious activities outside the home** | 0.08 |
| **Information Technology and Aging** | 0.06 | **Information Technology and Aging** | 0.08 |
| **Marriage** | 0.06 | **Frequency of hanging out with friends and neighbors** | 0.05 |
| **Outer wall material** | 0.06 | **No. of friends do you see or hear from** | 0.05 |
| **Attend religious activities outside the home** | 0.06 | **Marriage** | 0.05 |
| **Frequency of hanging out with friends and neighbors** | 0.05 | **How often is one of your friends available for you to talk to when you have an important decision to make** | 0.04 |
| **Educational attainment** | 0.04 | **Read the Bible or any religious materials** | 0.03 |
| **How often is one of your friends available for you to talk to when you have an important decision to make** | 0.04 | **Outer wall material** | 0.03 |
| **Roof material** | 0.03 | **Roof material** | 0.03 |
| **Wealth index** | 0.03 | **Wealth index** | 0.02 |
| **Perform religious activities at home with other family members** | 0.03 | **Type of housing** | 0.02 |
| **Read the Bible or any religious materials** | 0.03 | **Floor material** | 0.01 |
| **How often do you see or hear from friends** | 0.03 | **No. of friends do you feel at ease with** | 0.01 |
| **Type of housing** | 0.02 | **No. of friends do you feel close to** | 0.01 |
| **No. of friends do you feel close to** | 0.02 | **Educational attainment** | 0.01 |
| **Community type** | 0.02 | **Community type** | 0.01 |
| Floor material | 0.02 | **Living Arrangement of Older Person** | 0.01 |
| Watch or listen to religious activities through TV or radio | 0.02 | When one of your friends has an important decision to make, how often do they talk to you about it | 0.01 |
| Living Arrangement of Older Person | 0.02 | How often do you see or hear from friends | 0.01 |
| Living region | 0.02 | Frequency of gambling for leisure | 0.01 |
| When one of your friends has an important decision to make, how often do they talk to you about it | 0.01 | Pray by yourself or privately in places other than a public place of worship | 0.01 |
| Pray by yourself or privately in places other than a public place of worship | 0.01 | With/without insurance | 0.01 |
| Frequency of gambling for leisure | 0.01 | Perform religious activities at home with other family members | 0.01 |
| Tenure status | 0.01 | Watch or listen to religious activities through TV or radio | 0.01 |
| No. of friends do you feel at ease with | 0.01 | Living region | 0.01 |
| With/without insurance | 0.01 | Access to electricity | 0.01 |
| Access to electricity | 0.01 | Tenure status | 0.00 |

Note: Bold variables are selected by DeLong test.

**Figure S1.** The distribution of the polysocial score.

| 1. Histogram of polysocial score in men    |
| --- |
|  |
| 1. Histogram of polysocial score in women    |

**Figure S2.** LOWESS plot graphs displaying incidence of ADLs by polysocial score.

1. LOWESS plot in men

1. LOWESS plot in women

**Figure S3.** Agreement between observed and predicted prevalence of ADL disability for the continuous and categorical polysocial score.

| (a) Agreement plot among men based on continuous polysocial score  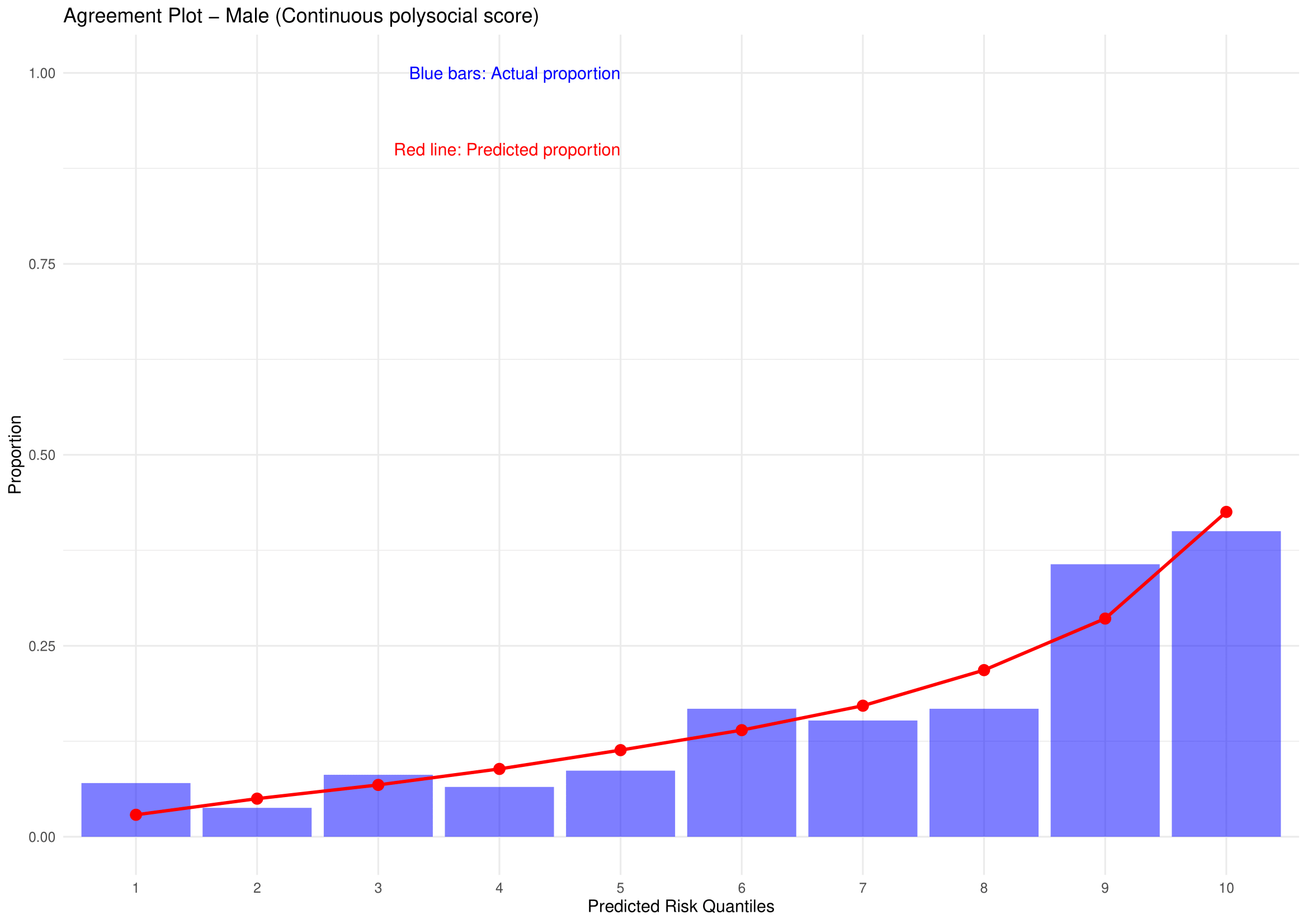 |
| --- |
| (b) Agreement plot among men based on categorical polysocial score  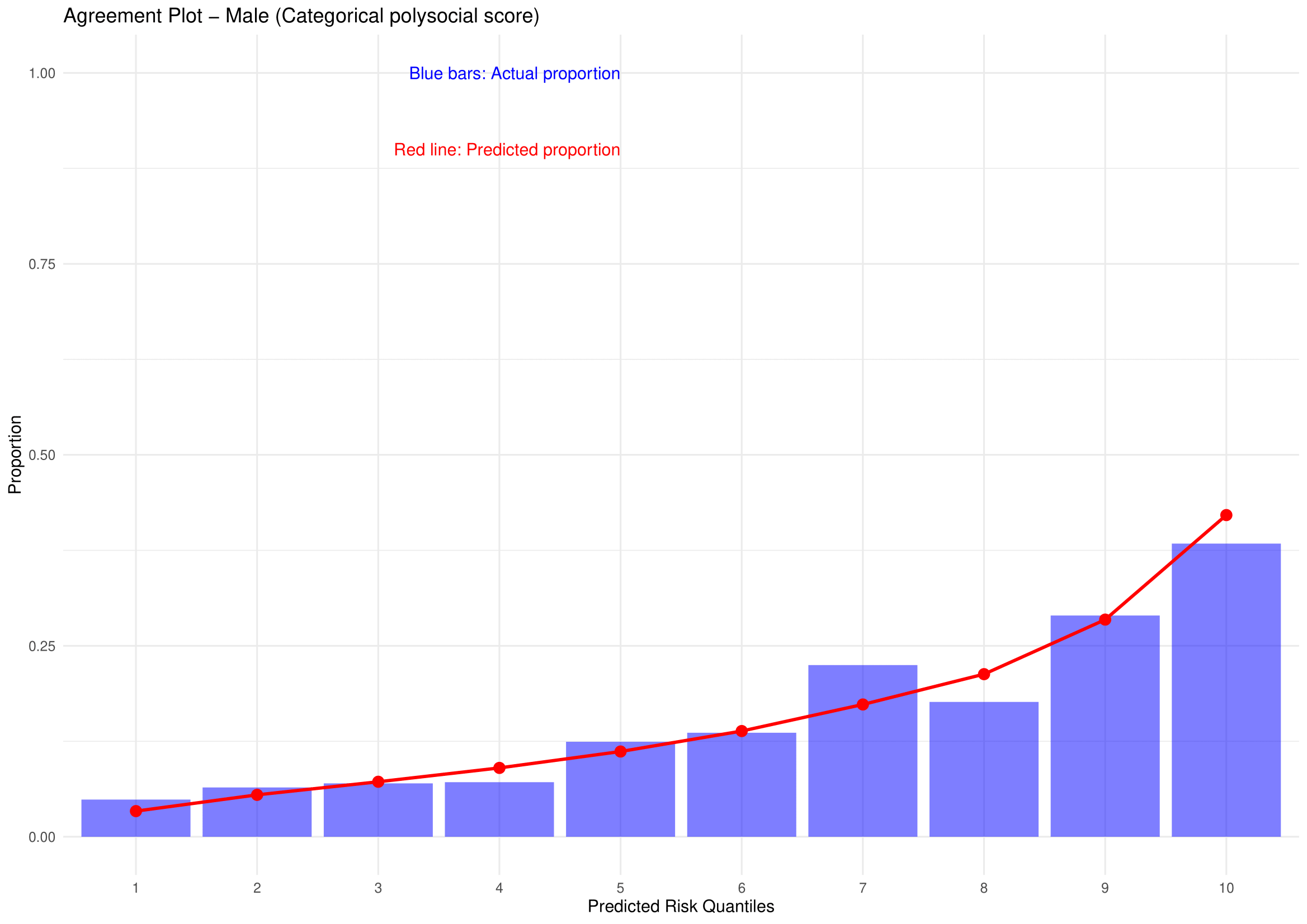 |
| (c) Agreement plot among women based on continuous polysocial score  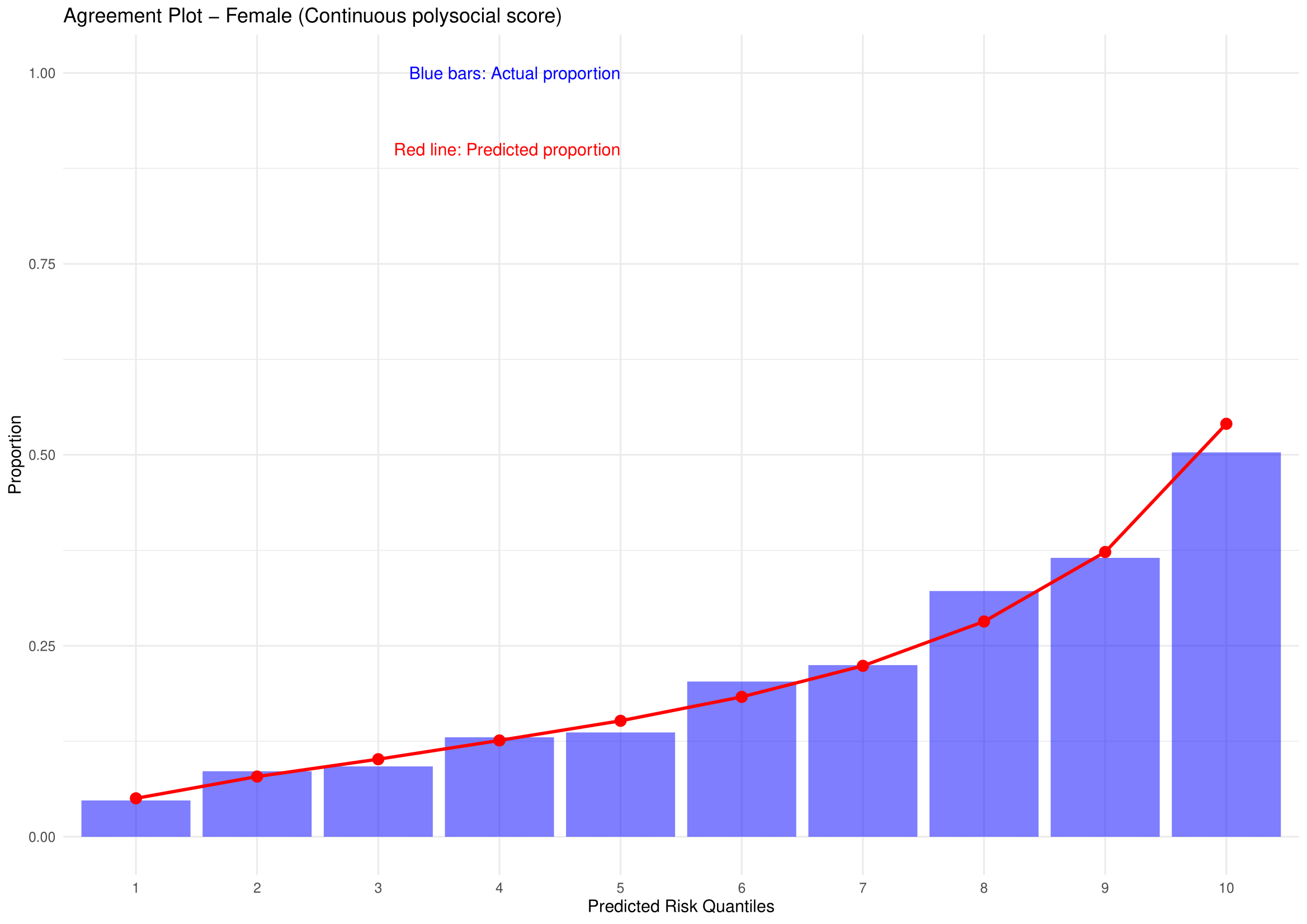 |
| (d) Agreement plot among women based on categorical polysocial score  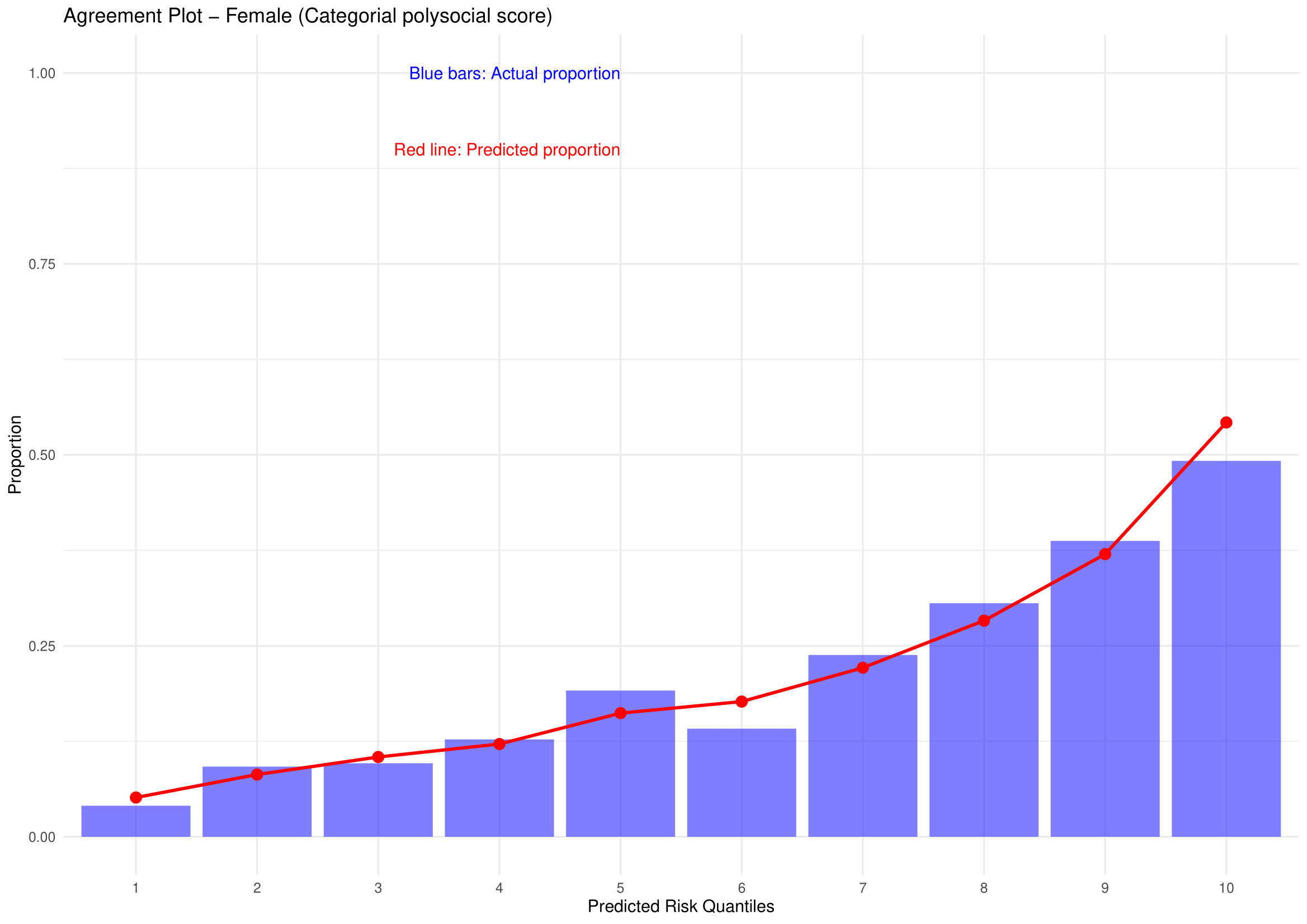 |
